# Gender differences in well-being among people living with non-communicable disease: The influence of social capital and grants

**DOI:** 10.1371/journal.pone.0337065

**Published:** 2025-12-01

**Authors:** Aaron Kobina Christian, Daniel Egerson, Sandra Boatemaa Kushitor

**Affiliations:** 1 Regional Institute for Population Studies, University of Ghana, Legon-Accra, Ghana; 2 Department of Community Health, Ensign Global University, Kpong, Ghana; 3 Department of Food Science and Centre for Sustainability Transitions, Stellenbosch University, Stellenbosch, South Africa; World Health Organisation: Organisation mondiale de la Sante, INDIA

## Abstract

**Background:**

This study explores how non-communicable diseases (NCDs), social capital, and government grants (social grants) influence subjective well-being (SWB) among individuals aged 40 and older in rural South Africa. Understanding gender differences in these relationships provides insights for improving public health interventions in resource-constrained settings.

**Methods:**

Data from 2,432 participants in the HAALSI Wave 3 study were analyzed to examine the predictors of SWB using regression models. Key covariates included age, education, marital status, employment, wealth, religion, social capital, and social grants. Interaction effects between NCDs, social capital, and social grants were evaluated, with gender-stratified analyses to explore disparities. SWB scores were computed, and statistical significance was assessed at various thresholds.

**Results:**

About a third of the sample had hypertension (58%), one-fifth had diabetes (20%), and nearly two-fifths had depression (36%). Having an NCD) was significantly associated with lower subjective wellbeing (β = −0.855, p < 0.001), with a slightly stronger negative effect observed among women than men. Older age (particularly 80+), and lower education were also associated with reduced wellbeing. Social capital did not moderate the negative impact of NCDs, as individuals with NCDs reported similarly low wellbeing regardless of high or low social capital. However, access to social grants showed some buffering effect: individuals with NCDs and high social grants reported better wellbeing outcomes compared to those with NCDs and low grants, particularly among males. Health insurance coverage was positively associated with wellbeing across all groups.

**Conclusions:**

These findings suggest that while NCDs significantly reduce wellbeing, social capital alone may not mitigate this burden, whereas targeted material support through grants may offer partial protection, particularly for men. We recommend the development of NCD financing strategies within the public healthcare funding schemes.

## Introduction

Non-communicable diseases (NCDs) emerge as a global public health concern, markedly in low- and middle-income countries (LMICs), where they account for 85% of premature deaths [1]. NCDs, including mental health conditions, are projected to incur a cumulative cost of US$ 21.3 trillion in LMICs over 2011–2030 [2]. According to the World Health Organization (WHO), sub-Saharan Africa (SSA) is expected to experience a 10% increase in mortality from NCDs between 2015 and 2030 [3]. Among the diverse range of NCDs, hypertension and diabetes are leading contributors to NCD-related mortality [4].

Given the established link between psychological well-being, health, and longevity [5] and evidence that individuals living with NCDs are more likely to have lower psychological well-being [6–8], identifying factors influencing well-being in this population is thus critical. This burden is evident not only in diverse global contexts, but also in community-based studies that shows that NCDs are strongly associated with diminished psychosocial wellbeing and quality of life [9]. Additionally, understanding gender differences is crucial within this narrative, as biological, socio-economic, and cultural factors create divergent pathways linking NCDs to well-being. Gender differences in psychological well-being are complex. While women often report higher emotional distress compared to men increasing their NCD risk [10], they tend to have stronger social support networks, which positively influence their well-being. Men, however, may experience higher levels of life satisfaction despite reporting more health risks or lower emotional expression, leading to inconsistent outcomes regarding gender and well-being [11]. Accounting for these disparities—which manifest unequally across contexts—is essential for designing equitable policies (SDG-3/SDG-10) that effectively address population-specific needs and reduce health inequalities [12].

Other factors that may influence the well-being of individuals living with NCD are social capital and social grants. Firstly, social capital, encompassing supportive relationships and community engagement, could provide emotional support, practical assistance, and a sense of belonging, all of which can enhance the well-being of those with NCDs. Although existing research has examined the impact of social capital on the well-being of individuals with NCDs [6,13], the findings have been inconsistent, and a gendered perspective remains largely absent. Consequently, the current study aims to investigate gender-specific differences in the relationship between non-communicable disease (NCD) status (hypertension, diabetes, and mental health conditions) and individual well-being. Furthermore, it seeks to assess how social capital and social grants moderates the association between NCD status and well-being.

We, therefore, contribute to the current scholarship in the following ways. First, we extend the discourse on non-communicable diseases (NCDs) by examining gender-specific differences in the relationship between NCD status (hypertension, diabetes, and mental health conditions) and individual well-being. This is important given that evidence on how such health outcomes interact with gender to shape well-being remains limited, particularly in the Global South. Significant gender differences exist in the manifestation and progression of NCDs, stemming from biological, social, and behavioral factors [14]. For example, although blood pressure is high among men, burden of hypertensive disorders is higher among women [15]. Consequently, examining gender-specific correlates of well-being among NCD patients is warranted to inform tailored interventions that address the distinct needs of each gender. Furthermore, gender roles and responsibilities critically influence chronic disease management diseases [16]. Women, particularly those from socio-economically disadvantaged backgrounds, frequently face heightened barriers to healthcare access, treatment adherence, and self-care. Gendered caregiving obligations and financial constraints often exacerbate these barriers among women [17]. Therefore, adopting a gender-sensitive approach to well-being analysis is essential for effectively identifying and addressing these systemic disparities.

Secondly, we add to the literature by analyzing the moderating role of social capital and social grants in the NCD–well-being nexus. Unlike studies that focus exclusively on biomedical or socio-economic determinants, our study highlights how social resources and institutional support structures shape the lived experiences of individuals with NCDs, thereby revealing underlying mechanisms. Social grants may mitigate inequities and enhance access to quality care by [1] raising the probability of affordability and [2] alleviating the financial strain on disposable income for treatment and illness management costs. In a recent study in Soweto, South Africa, respondent acknowledged that the management of NCDs such as diabetes and/or hypertension requires a level of care and engagement that requires money and time [18]. When examining the influence of subsidies or grants on the well-being of individuals with NCDs, it is critical to highlight gender differences and the differentiated factors that influence the well-being of men and women. This represents a crucial gap in the current body of scholarship, necessitating further investigation and understanding. Our third contribution is to the policy debate on inequality. We assess how current social policy and interventions affect well-being disparities across gender, building on existing work about welfare and health equity. We establish the impact of social grants on well-being in the context of NCDs, as has been reported for HIV/AIDS, food security and stunting. We analyze the effect of social grants on well-being related to NCDs [19]. Our findings provide robust evidence for financing NCDs through social interventions in LMICs where domestic and international NCD funding remain low. Using rich micro-level data and a multidimensional view of well-being, we provide robust evidence that strengthens research on health determinants in lower-income settings.

### Why South Africa

Over the past three decades, South Africa has experienced a ‘quadruple burden of disease’ consisting of the normalized yet troubling rates of HIV, high prevalence of tuberculosis, high levels of violence and injuries, consistently high maternal and child mortality rates, and the drastic upsurge in NCDs [20,21]. This is further compounded by high levels of uncontrolled NCD comorbidities [22–24] and metabolic syndrome [25].Yet, knowledge and awareness of NCD status is particularly low among men [26]. Unfortunately, concurrently, the chronicle of South Africa’s history of colonialism and Apartheid is tainted with social disintegration and destruction of social capital which has proven to affect health and general well-being, markedly among the black communities [27].

Social grants in South Africa are crucial in addressing poverty and inequality, particularly in a country still grappling with the legacies of historical injustices and socio-economic disparities [28]. Since independence, the government of South Africa has introduced and expanded social grants to all races [29]. These grants, which include child support, disability, and pensions, contribute to social security and improved health outcomes and cognitive functioning among beneficiaries, ultimately enhancing the well-being of entire communities [30]. In 2024, approximately 60 million people benefited from these grants [31,32]. Analysis of older adults who received these grants shows better cognitive health outcomes. Studies conducted in rural Mpumalanga reveal that cash transfer programs, such as the older person’s grant and the child support grant, significantly enhance memory function, reduce the risk of dementia, and lower mortality rates among recipients. As South Africa’s population continues to age, these findings highlight the importance of maintaining and investing in social grant programs, which can yield substantial benefits for vulnerable older adults [33].

Despite the above, studies examining the nexus between NCDs, social capital, and well-being are limited. For instance, Lau and Ataguba highlight that higher levels of social support and community engagement improve health outcomes for individuals with NCDs by fostering better chronic disease management [34]. Similarly, Campbell et al. emphasize that strong social networks significantly enhance mental health outcomes [35]. Christian et al. extend this understanding by demonstrating a positive association between social capital and subjective well-being among older adults with chronic diseases in low- and middle-income countries [6,34,35]. However, research exploring these relationships remains sparse, particularly from a gender perspective. By exploring these gaps, this study provides further nuanced insights into how NCDs affect male and female populations differently, offering more targeted policy recommendations for addressing gendered health disparities. Secondly, by examining how social capital and household wealth mitigate or exacerbate the negative effects of NCDs, this study provides valuable insights into potential mechanisms for improving well-being among individuals with chronic health conditions.

## Methods

### Study design, setting, and sample

This study used data from Wave 3 (2021–2022) of the Health and Aging in Africa Study: A Longitudinal Study of an INDEPTH Community in South Africa (HAALSI), conducted in rural Mpumalanga. HAALSI is a longitudinal cohort study (initiated in 2014) examining biological, social, and economic determinants of health and aging in adults aged ≥40 years drawn from the Agincourt Health and Demographic Surveillance System (HDSS). The Agincourt HDSS, active since 1992, systematically collects data on demographic events and key health, social, and economic indicators in the region. For Wave 3, 6,281 participants were randomly selected from the eligible HDSS population of 12,875, with 5,059 completing the baseline survey, resulting in an 80.5% response rate. The survey tools used for data collection were administered in the local language, Shangaan. The instruments were translated from English and then back translated to ensure reliability and accuracy.

### Ethical consideration

HAALSI received ethical approvals from the University of the Witwatersrand Human Research Ethics Committee (ref. M141159), the Harvard T.H. Chan School of Public Health, Office of Human Research Administration (ref. C13–1608–02), and the Mpumalanga Provincial Research and Ethics Committee. Participants were informed about the study and provided consent in xiTsonga or English. For those unable to read, a witness assisted, and consent was marked with an inked fingerprint. Autonomy and privacy were strictly upheld, and participants could withdraw at any time.

To minimize the risk of selection bias, the analytic sample was restricted to respondents (2,432 respondents) who were randomly assigned and completed questions related to subjective wellbeing and noncommunicable disease (NCD) status. Because these modules were administered based on random assignment within the overall survey design, the inclusion criteria are unlikely to have introduced systematic differences related to unobserved characteristics. This approach supports the assumption that the analytic sample is representative of the underlying study population. Furthermore, descriptive checks showed no substantial demographic or socioeconomic differences between those included and those excluded due to missing data on key variables.

Although Little’s MCAR test could not be performed due to insufficient degrees of freedom from sparse missingness patterns, descriptive comparisons between respondents with complete and incomplete data revealed no systematic differences in key demographic or health characteristics.

Ethical approval for the HAALSI study was obtained from the Harvard T.H. Chan School of Public Health, and all participants provided written informed consent following local regulations and institutional guidelines. HAALSI Public-use datasets can be accessed from https://doi.org/10.7910/DVN/Q2JFPV.

### Outcome variables

Subjective Well-being (SWB) encompassing both cognitive and emotional dimensions was computed as the outcome variable using data from the Wave 3 dataset. The Life Evaluation (cognitive well-being) was measured by averaging responses to questions such as overall life satisfaction and perceived life standing (e.g., “How satisfied are you with your life as a whole these days?”). Affect Balance (emotional well-being) was derived by calculating the difference between positive affect (e.g., “Did you smile or laugh a lot yesterday?”) and negative affect (e.g., “Did you experience stress yesterday?”). These scores were integrated to compute a total SWB score. The validity of subjective well-being (SWB) measures is well-established in both psychological and economic research, highlighting their reliability, cross-cultural relevance, and predictive capacity for health and economic outcomes. SWB indicators, such as life satisfaction and affective well-being scales, demonstrate high test-retest reliability, indicating consistent responses over time, even in the presence of situational fluctuations [36,37].

### Covariates

We included covariates based on prior research into well-being determinants: sex, age, education, marital status, wealth status, household size, employment status, health insurance, and religion [38–41]. Age was categorized into three groups: 40–59 years, 60–79 years, and 80 years and older. Other variables included marital status, employment status, educational attainment, household size (number of individuals living in the household), religion, and health insurance coverage, which was dichotomized as “Yes” or “No”. Additionally, a wealth index was derived through Principal Component Analysis (PCA) using data on household characteristics and asset ownership.

### Main explanatory variables

The primary explanatory variable in this study is the incidence of non-communicable diseases (NCDs), specifically focusing on hypertension, diabetes, and depression [42]. These conditions were selected due to their significant public health implications. Hypertension and diabetes are leading risk factors for severe complications, including cardiovascular diseases, kidney failure, and stroke, which collectively contribute to global morbidity and mortality [3]. Both conditions share modifiable lifestyle-related risk factors, such as obesity, unhealthy diets, and physical inactivity, underscoring the importance of effective management strategies [43]. Depression, on the other hand, exerts a profound influence on the management and outcomes of these chronic diseases. Individuals with depression often exhibit poor adherence to treatment protocols, heightening the risk of complications and adverse outcomes. Moreover, the bidirectional relationship between depression and other chronic conditions exacerbates health challenges, increasing healthcare costs and diminishing quality of life [44]. For this study, hypertension and diabetes were defined using the self-reported current treatment, with responses categorized as “Yes” or “No.” Depression was assessed using the Center for Epidemiologic Studies Depression Scale (CES-D) with a reliability Cronbach alpha co-efficient (α = 0.66) [45]. Respondents with scores of 16 or higher indicate a risk for clinical depression. This cutoff is a critical threshold for identifying individuals requiring further evaluation or intervention. Respondents meeting the criteria for any of these conditions were classified as having an NCD, while those not meeting the criteria were classified as not having an NCD. Several methodological safeguards were implemented to mitigate potential response bias due to mood-related fluctuations in self-reporting. First, the well-trained interviewers administered the CES-D scale in a structured manner to ensure consistency in responses. Second, efforts were made to establish rapport with participants to minimize social desirability bias and encourage honest reporting [46]. To minimize bias from self-reported depressive symptoms, the study validated the CES-D scale, which was tested explicitly for reliability among the aging Shangaan-speaking population in rural South Africa. Factor analyses confirmed its appropriateness in this cultural context, reducing measurement bias. Differential item endorsement by gender was assessed using the Mantel-Haenszel test to identify potential reporting differences. Additionally, the use of separate latent factors (Negative Affect and Diminished Positive Affect) allowed for a more nuanced analysis, helping to further control for bias in symptom reporting.

### Moderating variables

The moderating variables of interest were social capital and social grants. Social grants were measured using household government subsidies/grants. In contrast, social capital was computed in alignment with Social Capital Theory [47,48], which emphasizes the intrinsic value of relationships and social structures in providing access to resources, information, and opportunities. The social capital score comprised three components: social networks, social support, and social engagement with a reliability Cronbach alpha co-efficient (α = 0.75). Social networks were evaluated based on the number of networks, proximity to each network, the type of relationship, network strength (age composition), and frequency of interaction, with a composite score calculated by summing these dimensions [49,50]. Social support was measured using an egocentric network approach, capturing the frequency of emotional, physical, informational, and financial support provided by up to seven key individuals in the respondent’s network over the past six months (See Table 1).

**Table 1:** Computing social capital index.

Dimension	Components	Scoring Criteria	Maximum Score
Social Network	1. Number of Networks	1 point per listed network (max 7 networks)	7
	2. Proximity to Networks	Household (5), Same village (4), Within Agincourt (3), South Africa (2), Abroad (1)	35
	3. Nature of Relationship	Married/Cohabiting (4), Relatives (3), Friends (2), Others (1)	28
	4. Age of Network	18–35 years (5), 36–50 years (4), 51–65 years (3), 66–75 years (2), 76 + years (1)	35
	5. Frequency of Interaction	Daily (6), Few times/week (5), Weekly (4), Few times/month (3), Monthly (2), Rarely (1), Never (0)	42
Total Social Network Score	Summation of all 5 components	**147**	
Social Support	1. Emotional Support	6 (daily) to 0 (none), summed across 7 networks	42
	2. Financial Support	6 (daily) to 0 (none), summed across 7 networks	42
	3. Physical Support	6 (daily) to 0 (none), summed across 7 networks	42
	4. Informational Support	6 (daily) to 0 (none), summed across 7 networks	42
Total Social Support Score	Summation of all 4 support types	**168**	
Social Engagement	Participation in Activities	1 point per activity completed (max 10 activities)	10
Overall Social Capital Score	Sum of Social Network, Social Support, and Social Engagement	**326**	
Classification	High/Low Social Capital	Above or below 50th percentile	–

Each type of support was scored based on contact frequency, with responses aggregated into a total monthly support score. Social engagement was assessed by the number of activities (out of 10) the respondents participated in within the last month [51]. The overall social capital score was derived by summing the scores for social networks, support, and engagement, with a maximum possible score of 326, categorized into high or low social capital based on the 50th percentile (163).

The second moderating variable, household government subsidies or grants, was assessed by documenting the receipt of any state-provided support. Thirteen different types of grants were listed, including old-age grants, child support grants, unemployment insurance, war veteran pensions, social relief or distress grants etc. While there are tendencies for gender disparities in accessing these grants, which could be better explored through a qualitative study, our analysis was limited to the available quantitative data. For analytical purposes, this was further recategorized as high (two or more grants) and low (zero to one grant). These measures allowed for a more comprehensive assessment of the moderating influences on the study’s outcomes.

### Statistical analysis

Data management, pre-processing, and analysis were conducted using STATA 17 (StataCorp, USA) and R Package 4.3.1. All respondents with complete data on the primary independent variables, outcome variables, and covariates were included in the analysis. Descriptive statistics for all variables of interest were generated and presented as frequencies and proportions for categorical variables.

### Regression analysis

The primary model employed an OLS function to analyze the relationship between subjective well-being (the outcome variable) and the incidence of non-communicable diseases (NCDs) as the main independent variable, alongside a set of covariates. The model is specified in Equation (1):


$$\begin{array}{cc} NCDStatus= & \beta_{\text{0}}+\beta_{\text{1}}NCD+\beta_{\text{2}}Age+\beta_{\text{3}}Education+\beta_{\text{4}}MaritalStatus\\ & +\beta_{\text{5}}Religion+\beta_{\text{4}}MaritalStatus+\beta_{\text{5}}Wealth\\ & +\beta_{\text{6}}Householdsize+\beta_{\text{6}}HealthInsurance \end{array}$$ (1)

Two separate models were run for males and females. In this model, p represents the probability of improvements in subjective well-being, and β_0_, β_1_, β_2_,…,β_8_ are the coefficients for the intercept, NCD incidence, and the other explanatory variables, including age, formal education, marital status, religion, household wealth, household size, and health insurance. Beta coefficients were chosen over odds ratios for their clearer interpretation of effect sizes and directions. Marginal effects were also calculated to illustrate how a one-unit change in an independent variable affects the predicted probability of the outcome, providing a more intuitive understanding of the results.

### Moderation analysis

We explored the potential moderating effects of social capital and social grants on the relationship between NCD incidence and subjective well-being. This interaction effect was modeled by incorporating interaction terms for NCDs and social capital, as


$$\begin{array}{cc} Wellbeingscore= & \beta_{\text{0}}+\beta_{\text{1}}(NCD*SocialCapital/socialgrants)+\beta_{\text{2}}Age+\beta_{\text{3}}Education\\ & +\beta_{\text{4}}MaritalStatus+\beta_{\text{5}}Religion+\beta_{\text{4}}MaritalStatus+\beta_{\text{5}}Wealth\\ & +\beta_{\text{6}}Householdsize+\beta_{\text{6}}HealthInsurance \end{array}$$ (2)

well as NCDs and social grants, into the regression equation, as shown below:

Where:

p: Probability of high subjective well-being.; β_0_: Intercept.; β_1_, β_2_, β_3:_ Main effects of NCD, Social Capital and Social Grants, respectively. β_4_, β_5_: Interaction effects of NCD with Social Capital and social grants; ∑^n^_i=6_ βi- Covariates: Effects of other covariates included in the model (e.g., age, sex, employment status, education level, etc.). This model evaluates the direct and moderating effects of Social Capital and social grants on the relationship between NCDs and subjective well-being, while controlling for other variables. All analyses were conducted using STATA 17. Regression diagnostics were performed to assess key model assumptions. Multicollinearity was evaluated using variance inflation factors (VIF), with all values falling below 5. Heteroscedasticity was assessed using the Breusch–Pagan test, which indicated no significant violations. Residual plots were also examined to confirm linearity and normality assumptions.

**Robustness test:** This study further applies the Kinky Least Squares (KLS) regression to test the robustness of the estimated impact of non-communicable diseases (NCDs) on subjective well-being (SWB), especially under the possibility of endogeneity. KLS is particularly useful when valid instrumental variables are unavailable, or exclusion restrictions are difficult to justify [52]. In our setting, endogeneity may arise from bidirectional causality, where poor health lowers well-being, but lower well-being also increases disease risk, as well as from unobserved confounders such as latent health behaviors or psychosocial stressors that simultaneously affect both NCD status and SWB. Unlike conventional IV methods, which attempt to remove endogeneity by introducing external instruments, KLS explicitly incorporates the endogeneity into estimation. The method corrects the bias of OLS by considering the possible correlation between the endogenous regressor (NCD) and the regression error term. Researchers specify a plausible range for this correlation, referred to as the “kink,” which reflects prior knowledge or assumptions about the likely strength and direction of endogeneity. For each value within this range, KLS produces a corrected coefficient estimate, and the union of these results yields confidence intervals that remain valid even when endogeneity is present.

In our analysis, we specified a correlation of 0.5 between NCD and the error term, representing a moderate degree of endogeneity consistent with the hypothesized feedback relationship. The KLS estimates under this assumption remain close to the baseline results, and the confidence intervals continue to exclude zero. This provides reassurance that our main finding, the negative association between NCDs and SWB is not an artifact of reverse causality or omitted confounders but rather reflects a robust empirical relationship.

## Results

### Background characteristics

The analytic sample for the current study included 2,432 respondents, with a higher proportion of females (59.3%) than males (40.7%). Employment levels were low, with 90.7% of participants reporting that they were unemployed. Regarding educational attainment, 42% had no formal education, 37.2% had completed primary education, and 20.9% had attained secondary or higher education. Nearly half (48.9%) of respondents were ever married, while 45.2% were currently married or cohabiting, and 5.9% were never married. The prevalence of hypertension was 58%, diabetes was 20%, and 36% had depression (36%). A significant proportion (75.9%) of respondents reported living with a non-communicable disease (NCD). For wealth, 38.7% were classified as poor, 19.6% as middle-income, and 41.7% as rich. For respondent age, 30.3% were under 60 years, 54.9% between 60 and 79, and 14.8% aged 80 or older. Despite the widespread prevalence of health challenges, only 11% were enrolled in a health scheme, underscoring a gap in healthcare access within the population

Table 2. provides a detailed description and statistical summary of the variables used in this study by the gender of the respondent. We observed statistically significant sex-based differences across several sociodemographic and health-related variables.

**Table 2:** Distribution of select demographic and socioeconomic variables by gender of respondent.

Variable	Women		Men		*P-value*
	*Percent*	*Count*	*Percent*	*Count*	
**Employment Status**					0.007
Not Employed	92.03	1,328	88.78	878	
Employed	7.97	115	11.22	111	
**Education Level**					<0.001
None	45.25	653	37.21	368	
Primary	35.90	518	39.03	386	
Secondary +	18.85	272	23.76	235	
**Marital Status**					<0.001
Never Married	5.13	74	6.98	69	
Married/Living with Partner	31.32	452	65.42	647	
Ever Married	63.55	917	27.60	273	
**Wealth Quintile**					0.002
Poor	35.76	516	42.87	424	
Middle	20.58	297	18.20	180	
Rich	43.66	630	38.93	385	
**Religion**					<0.001
None	2.70	39	17.09	169	
Christianity	94.25	1,360	74.42	736	
African Traditional	3.05	44	8.49	84	
**Age Group**					0.004
<60	32.78	473	26.69	264	
60-79	53.36	770	57.03	564	
80+	13.86	200	16.28	161	
**Health Scheme Coverage**					0.357
No	88.50	1,277	89.69	887	
Yes	11.50	166	10.31	102	
**Hypertension diagnosis**					0.059
No	40.54	585	44.39	439	
Yes	59.46	858	55.61	550	
**Diabetes diagnosis**					0.072
No	78.93	1,139	81.90	810	
Yes	21.07	304	18.10	179	
**Depression**					0.607
No	64.52	931	63.50	628	
Yes	35.48	512	36.50	361	
**Incidence of NCDs**					0.650
No	23.77	343	24.57	243	
Yes	76.23	1,100	75.43	746	
**Social grants received**					0.045
Low	94.73	1,367	96.46	954	
high	5.27	76	3.54	35	
**Social Capital**					0.048
Low	29.87	431	26.19	259	
High	70.13	1,012	73.81	730	
	*Mean*	*Std. Dev.*	*Mean*	*Std. Dev.*	
**Subject well-being score**	9.20	2.24	9.20	2.31	0.9712

A higher proportion of women were not employed compared with men (92.03% vs 88.78%, p = 0.007), and fewer women attained secondary or higher education (18.85% vs 23.76%, p < 0.001). Marital status varied markedly by sex: women were more likely to have ever been married (63.55% vs 27.60%), whereas men were predominantly married or living with a partner (65.42% vs 31.32%, p < 0.001). Men were more likely to identify with no religion (17.09% vs 2.70%, p < 0.001) and more frequently belonged to the poorest wealth quintile (42.87% vs 35.76%, p = 0.002). Differences were also apparent in age distribution, with a greater proportion of men aged 80 years or older (16.28% vs 13.86%, p = 0.004). Moreover, women were marginally more likely to receive high levels of social grants (5.27% vs 3.54%, p = 0.045) and to report low social capital (29.87% vs 26.19%, p = 0.048). No significant differences were observed in health insurance coverage, diagnoses of hypertension or diabetes, depression, or incidence of non-communicable diseases

### Association between non-communicable diseases and Subjective well-being

**Fig 1:**
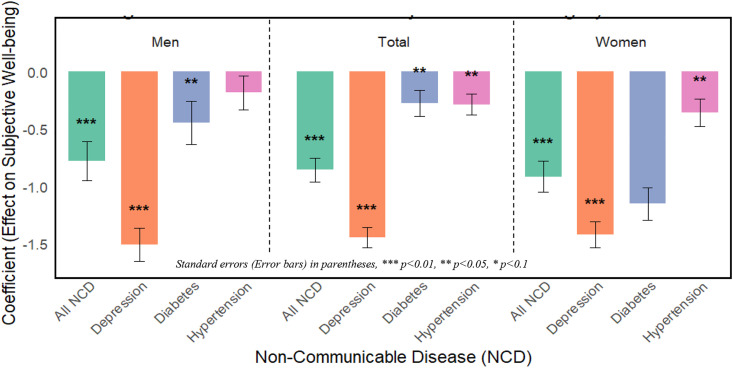
Effect of NCDs on Subjective Well-being by Gender.

Overall Non-communicable diseases (NCDs) significantly reduced subjective well-being (SWB) (Table 3). This negative effect was stronger for women (−0.914*) than for men (−0.778*), indicating women experience a greater psychological burden from cumulative chronic health conditions. Depression had the strongest negative impact on subjective well-being (SWB) for both men and women. Its effect was larger for men (−1.510*) than for women (−1.420*) (Fig 1).

**Table 3:** Association between NCD incidence and subjective well-being.

Non communicable disease (Ref: None)	Total			Women			Men		
	Coeff.	SE	95% CI	Coeff.	SE	95% CI	Coeff.	SE	95% CI
Yes	−0.855***	(0.104)	−1.051 to 0.649	−0.914***	(0.134)	−1.176 to −0.652	−0.778***	(0.168)	−1.108 to −0.449
Age group (Ref: 40–59 yrs)									
60-79	−0.257**	(0.111)	−0.473 to 0.039	−0.266*	(0.140)	−0.541 to 0.009	−0.199	(0.188)	−0.569 to 0.170
80+	−1.212***	(0.155)	−1.519 to −0.910	−1.257***	(0.206)	−1.661 to −0.853	−1.129***	(0.249)	−1.618 to 0.640
Formal education (Ref: None)									
Primary	0.061	(0.103)	−0.141 to 0.263	0.102	(0.131)	−0.155 to 0.359	0.011	(0.168)	−0.341 to 0.320
Secondary +	0.400***	(0.135)	0.135 to 0.664	0.544***	**(0.184)**	0.183 to 0.905	0.207	(0.203)	−0.191 to 0.604
Marital status (Ref: Never married)									
Living with partner	0.629***	(0.200)	0.237 to 1.020	0.365	(0.272)	−0.168 to 0.899	0.903***	**(0.301)**	**0.312 to 1.493**
Ever married	0.287	(0.047)	−0.099 to 0.674	0.049	(0.264)	−0.471 to 0.565	0.567*	**(0.304)**	**−0.0287 to 1.163**
Employment status (Not employed)									
Employed	0.246	(0.161)	−0.070 to 0.562	0.231	(0.217)	−0.196 to 0.657	0.278	(0.249)	−0.211 to 0.767
Household wealth (Ref: Low)									
Middle	0.311**	(0.125)	0.066 to 0.555	0.389**	(0.157)	0.080 to 0.697	0.189	(0.205)	−0.214 to 0.591
Rich	0.043	(0.108)	−0.168 to 0.254	−0.014	(0.137)	−0.283 to 0.254	0.128	(0.176)	−0.219 to 0.474
Household size	0.021	(0.053)	−0.082 to 0.124	0.049	(0.070)	−0.089 to 0.187	0.007	(0.080)	−0.178 to 0.151
Religion (Ref: No religion)									
Christianity	−0.039**	(0.165)	−0.717 to −0.069	−0.600*	(0.346)	−1.279 to 0.079	−0.365*	(0.195)	−0.748 to 0.018
African Traditional	−0.269	(0.245)	−0.749 to 0.211	−0.737	(0.468)	−1.65 to 0.181	−0.091	(0.302)	−0.684 to 0.502
Has health insurance (Ref: No)									
Yes	0.577***	(0.142)	0.299 to 0.855	0.390**	(0.177)	0.042 to 0.738	0.854***	**(0.236)**	**0.391 to 1.317**
Gender (Ref: Women)									
Men	−0.155	(0.103)	−0.357 to 0.048						
Observations	2432			1443			989		
R-squared	0.101			0.116			0.089		

Value represents coefficients and Standard errors are in parentheses, *** p < 0.01, ** p < 0.05, * p < 0.

The impact of specific non-communicable diseases (NCDs) on subjective well-being (SWB) varied by gender. Hypertension significantly lowered SWB in women (−0.355) but had no significant effect in men (−0.182), suggesting women may be more vulnerable to cardiovascular-related stressors. In contrast, diabetes had a large (but non-significant) negative effect on women (−1.152), while its impact on men was smaller yet statistically significant (−0.445) (Fig 1)

### Robustness analysis

Table 4 presents the results from a Kinky Least Squares (KLS) regression model estimating the effect of NCDs on subjective well-being, accounting for potential endogeneity.

**Table 4:** The effect of NCD on well-being using Kinky Least Squares regression.

Variable	Coefficient	Std. Err.		95% CI
NCD	−3.892	0.158	***	−4.203 to −3.582
Covariate Present	*YES*	*YES*		
Postulated endogeneity	−0.5			
_cons	10.689	0.430113		9.845 to 11.532

* ** p < 0.01, * * p < 0.05, * p < 0.1

The results show that the negative effect of NCD on subjective well-being becomes substantially larger under this assumption, with the estimated coefficient increasing in magnitude in the standard OLS model to –3.89 using the KLS estimator. This suggests that failing to account for endogeneity likely understates the true burden of NCDs on individual well-being. In other words

the KLS analysis provides strong evidence that the negative association between NCD and well-being is robust to moderate levels of endogeneity.

### Association between other covariates and Subjective well-being

Educational attainment was positively associated with well-being; possessing secondary education or higher significantly increased the predicted log-odds of higher well-being compared to having no formal education (β = 0.400, p < 0.01). Marital status also exerted a positive influence, with individuals currently married or cohabiting reporting significantly higher well-being (β = 0.629, p < 0.01). Within the wealth quintiles, membership in the middle quintile was associated with a significant increase in well-being relative to the reference group (β = 0.311, p < 0.05). Finally, religious affiliation showed a significant negative association, as Christian identification was linked to lower well-being (β = −0.393, p = 0.017)

### Gender differences in Well-being determinants

The analysis of well-being determinants revealed significant gender differences in the factors influencing individual well-being, particularly in relation to education, marital status, and health insurance coverage. Among females having secondary or higher significantly improves their well-being (0.544, p < 0.01) compared to those with only primary education. This was not so among the men. In contrast, the effect of marital status on well-being was more significant for males than females. While being married or living with a partner was associated with better well-being for both genders, the positive impact was notably stronger for males (both living with partner and ever married and for women only for those living with partner), indicating that marriage or partnership might provide greater social support or stability for men.

### Moderation analysis

The analysis investigates the moderating role of social capital and social grants in the relationship between NCDs and well-being, while controlling for other demographic and socioeconomic factors.

*A. NCD and Social Capital Interaction*: In the overall sample, individuals with NCDs reported significantly lower levels of well-being compared to those without NCDs, regardless of their level of social capital (Table 5). Specifically, compared to individuals without NCDs and with low social capital (the reference group), those with NCDs and low social capital had a significantly lower well-being score (β = −0.899, SE = 0.188, p < 0.01), as did those with NCDs and high social capital (β = −0.944, SE = 0.176, p < 0.01). In contrast, individuals without NCDs but with high social capital did not differ significantly in well-being from the reference group (β = −0.113, SE = 0.197, p > 0.05).

**Table 5:** Moderation effect of Social Capital and Social grants on the well-being of individuals with NCDS by Gender.

	All Sample		Women		Males	
SOCIAL CAPITAL (Ref: No NCD & Low Social Capital)						
No NCD with High Social Capital	−0.113	(0.197)	0.053	(0.250)	−0.356	(0.324)
NCD with Low Social Capital	−0.899**	(0.188)	−0.818 **	(0.237)	−1.041**	(0.309)
NCD with High Social Capital	−0.944**	(0.176)	−0.902**	0.222	−1.022**	(0.290)
Other covariate Present	Yes		Yes		Yes	
**Grants** (Ref: No NCD & High grants)						
No NCD with High Social grants	−0.059	(0.394)	0.396	(0.478)	−1.056	(0.690)
Has NCD with Low Social grants	−0.872***	(0.107)	−0.891***	(0.138)	−0.865***	(0.171
Has NCD with High Social grants	−0.558**	(0.261)	−0.892**	(0.310)	0.236	(0.478)
Other Covariate Present	Yes		Yes		Yes	

Value represents coefficients and Standard errors are in parentheses, *** p < 0.01, ** p < 0.05, * p < 0.1. Other Covariates: Covariates controlled for in Table 2 *above.*

Gender-stratified analyses revealed consistent patterns across males and females. Among females, having an NCD was associated with significantly lower well-being, both for those with low social capital (β = −0.818, SE = 0.237, p < 0.01) and high social capital (β = −0.902, SE = 0.222, p < 0.01), relative to the reference group. A similar trend was observed among males: the well-being of those with NCDs and low social capital was significantly lower (β = −1.041, SE = 0.309, p < 0.01), as was that of those with NCDs and high social capital (β = −1.022, SE = 0.290, p < 0.01). In both gender groups, individuals without NCDs but with high social capital showed no significant difference in well-being compared to the reference group. These findings underscore that the adverse impact of NCDs on well-being is consistent across gender and not substantially moderated by social capital. Overall, the interaction between NCDs and social capital appears to be stronger for men than for women. This may reflect the greater importance typically placed on social capital by men in developing economies, where social networks and community ties often play a more central role in their social well-being. Consequently, men with either low or high social capital and NCDs experience a comparatively greater reduction in well-being.

*B: NCD and social grants Interaction*: In the overall sample, individuals with NCDs consistently reported significantly lower levels of well-being than those without NCDs, and this relationship varied depending on access to social grants. Compared to individuals without NCDs and without social grants (reference group), those with NCDs and no social grants had substantially lower well-being (β = −0.872, SE = 0.107, p < 0.001). However, individuals with NCDs who received social grants also reported significantly reduced well-being (β = −0.558, SE = 0.261, p < 0.01), though the negative effect was notably smaller in magnitude. Among individuals without NCDs, receiving social grants was not significantly associated with well-being (β = −0.059, SE = 0.394, p > 0.05). These findings suggest that while having an NCD is associated with lower well-being, receipt of social grants may buffer some of the negative effects associated with NCD.

Gender-disaggregated results showed similar patterns with subtle differences in the strength of associations. Among females, having an NCD and no social grants was strongly associated with lower well-being (β = −0.891, SE = 0.138, p < 0.001). For females with NCDs who received social grants, the negative association with well-being remained significant (β = −0.892, SE = 0.310, p < 0.01), but the magnitude was comparable to that of those without grants, suggesting limited moderating impact. In contrast, among males, having an NCD without access to grants also led to significantly lower well-being (β = −0.865, SE = 0.171, p < 0.001), but males with NCDs who received grants showed no significant difference in well-being from the reference group (β = 0.236, SE = 0.478, p > 0.05). This indicates that social grants may play a stronger protective role for males with NCDs compared to females, potentially offsetting the well-being disadvantage associated with NCDs in men.

**Fig 2:**
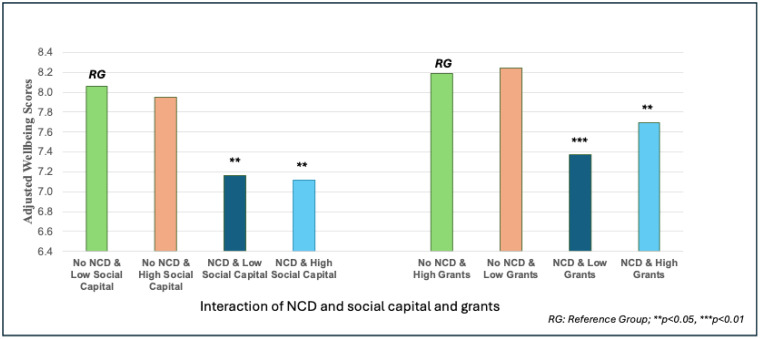
Moderating effect of social capital and social grants on Well-being score.

Briefly, Fig 2 shows that, as discussed, individuals without NCDs reported higher adjusted subjective well-being scores across both social capital and social grants groups.

Among those with NCDs, receiving higher social grants was associated with a modest but statistically significant improvement in well-being, whereas social capital showed a limited moderating impact.

## Discussion

This study examined the relationship between non-communicable diseases (NCDs) and individual well-being, and the moderating roles of social capital and social grants. Our findings demonstrate that individuals with NCDs report significantly lower well-being than those without chronic conditions, consistent with extensive literature documenting the adverse physical, psychological, and social impacts of NCDs like diabetes, hypertension, and cardiovascular disease on quality of life [53,54].

Notably, NCDs were associated with reduced well-being irrespective of social capital levels. Although social capital is widely regarded as an important determinant of well-being, offering emotional support, access to information, and resilience in the face of adversity [55], its capacity to buffer the negative impact of NCDs appears limited. Prior studies suggest that while social capital enhances coping mechanisms and promotes better psychological outcomes, the persistent functional limitations, stigma, and financial strain associated with NCDs often overwhelm these protective effects [56]. In this study, individuals living with NCDs reported similarly reduced levels of well-being regardless of whether they had high or low levels of social capital, suggesting that the protective influence of social networks may be insufficient to offset the burden of chronic illness. Thus, the protective effect of social capital may be attenuated by persistent stressors and functional limitations inherent to chronic conditions. Alternatively, NCD-related physical or emotional constraints may constrain individuals’ ability to leverage social networks for a meaningful effect on their well-being. Additionally, because of the high levels of caregiver burden, social support systems do not always endure in the face of adversity [57].

Conversely, social grants significantly moderated the NCD-well-being relationship. The study finding aligns with the work of Fadzil et al., who concluded that financial assistance to older individuals significantly enhances their well-being, acting as a buffer during stressful situations such as managing an NCD [58]. This buffering effect, particularly pronounced among men, suggests financial transfers alleviate economic strain associated with chronic illness. Prior evidence confirms cash transfers reduce vulnerability by improving healthcare access, mitigating financial hardship, and enhancing psychological well-being [59]. The weaker effect among women may reflect disproportionate caregiving burdens, social role constraints, or inadequate grant value relative to household needs, indicating financial support alone cannot address their multifaceted responsibilities. In South Africa, social grant recipients are not likely to have private health insurance and private healthcare. Therefore, access to social grants can provide an opportunity for individuals with NCDs to finance their out-of-pocket payments associated with the subsidized public healthcare system [60].

While the wealth-well-being relationship merits further investigation, our findings underscore that poverty reduction (low to middle wealth) is vital for enhancing well-being in NCD patients, whereas wealth maximization (low to rich) yields diminishing returns. This threshold pattern aligns with global evidence showing wealth improves health outcomes primarily through poverty alleviation, as medical costs from severe NCDs erode well-being gains even at higher wealth levels [61]. Consequently, interventions should prioritize lifting the poorest NCD patients out of poverty through safety nets, not generalized wealth promotion.

Generally, the interaction NCDs and social grants demonstrates that financial support can significantly improve the well-being of individuals with NCDs. Social grants are essential for meeting immediate household needs, but for many South African households, they often become the primary source of income, creating a dependency that may lead to unintended long-term consequences [62]. This notwithstanding given the peculiar needs of the elderly with NCDs the provision is crucial.

Furthermore, gender disparities emerge regarding how social grants impact well-being. Women with NCDs consistently report lower well-being across various grant/subsidies levels, underscoring the need for gender-sensitive policies. While higher social grants help mitigate some adverse effects of NCDs for both genders, women appear to derive more significant benefits from such support. Previous research emphasizes that women’s health outcomes are profoundly shaped by their socio-economic status and access to resources, indicating that health interventions must comprehensively address these underlying dynamics [63]. These insights necessitate the implementation of gender-sensitive policies focused on addressing unique gender needs can enhance well-being outcomes for marginalized populations [64]. The observed pattern of social capital being less protective for men may be due to gendered support-seeking behaviors. Prior research indicates men often derive less psychological benefit from networks, as masculine norms can discourage help-seeking [65,66]. Additionally, standard social capital measures may not capture functional support relevant to men during illness. These findings highlight the need for gender-sensitive interventions leveraging social capital in chronic disease management

### Limitations of the study

This study utilised data from the most recent and comprehensive wave of the HAALSI cohort (Wave 3) to investigate how noncommunicable diseases (NCDs), social capital, and social grants intersect to shape subjective well-being among adults aged 40 years and older in rural South Africa. While the cross-sectional design provides valuable insight into these associations, it limits causal inference and the ability to assess change over time. Future longitudinal analyses across multiple waves of HAALSI would be essential to capture temporal trends better and identify potential causal pathways. Additionally, although social capital is widely recognised as a critical determinant of well-being, particularly for its potential to buffer the negative impacts of chronic illness, its protective effect may be limited in some contexts. This study operationalized social capital through structural indicators (i.e., the extent of individuals’ engagement in social networks). However, this approach may not capture distinct types of social capital and the quality or functional utility of those connections, such as the emotional support or practical assistance people receive when managing illness. As such, future research should incorporate more nuanced measures of social capital that reflect both structural and functional dimensions, to more accurately assess its potential role in mitigating well-being losses among individuals living with NCDs. Future studies could explore the examined relationships with other NCDs for better attempt to generalizability. Additionally, social grants were measured at the household level and attributed to individuals, which may not capture intra-household dynamics or gendered control over resources. Future studies with individual-level data may be better suited to examine these relationships.

## Conclusion

The implications of this study extend well beyond the immediate context of South Africa, offering valuable insights pertinent to developing countries grappling with the intertwined challenges of non-communicable diseases (NCDs), social capital, and gender disparities. By highlighting the role of social capital and social grants in shaping individual well-being, this research underscores the necessity for holistic public health strategies that consider the unique socio-economic factors influencing diverse populations, as outlined in South Africa’s National Health and Social Development Policy. Addressing such interrelated issues is not merely a matter of health policy but a broader commitment to fostering equitable socio-economic environments that promote health equity, particularly for vulnerable groups such as women. Policymakers must recognize the systemic barriers that impede access to resources and support networks, thereby shaping culturally sensitive and contextually appropriate initiatives for diverse communities. Designing NCD financing strategies within the public healthcare funding schemes is crucial.

To drive meaningful change, a multifaceted approach is required. This should encompass the development of gender-sensitive policies to rectify disparities in access to healthcare and resources. While capacity-building programs tailored specifically for women remain essential for empowering them to navigate their healthcare journeys effectively, it is equally important to address barriers that limit men’s engagement with healthcare services. Promoting health-seeking behavior among men, establishing male-friendly healthcare services, and implementing community-based education programs targeted at men can enhance their engagement with preventive healthcare and early NCD management.

Furthermore, enhancing community support structures is crucial in fostering social capital, as strong community ties can significantly bolster resilience against chronic health challenges. By implementing these strategies, developing countries can construct a more supportive infrastructure that not only improves health outcomes for marginalized populations but also paves the way for sustainable socio-economic advancement. Prioritizing these interventions will ultimately contribute to creating healthier, more resilient communities and addressing the broader determinants of health in a sustainable manner.
